# Single-Cell Multiomics: Multiple Measurements from Single Cells

**DOI:** 10.1016/j.tig.2016.12.003

**Published:** 2017-02

**Authors:** Iain C. Macaulay, Chris P. Ponting, Thierry Voet

**Affiliations:** 1 Earlham Institute, Norwich Research Park, Norwich NR4 7UH, UK; 2 Sanger Institute – EBI Single-Cell Genomics Centre, Wellcome Trust Sanger Institute, Hinxton CB10 1SA, UK; 3 MRC Human Genetics Unit, MRC IGMM, University of Edinburgh, Crewe Road, Edinburgh EH4 2XU, UK; 4 Department of Human Genetics, University of Leuven, KU Leuven, Leuven, 3000, Belgium

**Keywords:** epigenomics, genomics, multiomics, proteomics, single cell, transcriptomics

## Abstract

Single-cell sequencing provides information that is not confounded by genotypic or phenotypic heterogeneity of bulk samples. Sequencing of one molecular type (RNA, methylated DNA or open chromatin) in a single cell, furthermore, provides insights into the cell's phenotype and links to its genotype. Nevertheless, only by taking measurements of these phenotypes and genotypes from the same single cells can such inferences be made unambiguously. In this review, we survey the first experimental approaches that assay, in parallel, multiple molecular types from the same single cell, before considering the challenges and opportunities afforded by these and future technologies.

## Trends

Unambiguous inference that a cellular phenotype is caused by a genotype can only be achieved by their measurement from the same single cell.

Estimating RNA and DNA copy number abundance in single cells is now possible using a variety of experimental approaches.

Parallel measurement of single-cell epigenomes and transcriptomes provides further insight into the regulation of cellular identity and phenotypes.

Parallel measurement of single-cell transcriptomes and protein abundance (by FACS, proximity ligation assays/PEA or mass cytometry) allows insight into expression dynamics.

Our understanding of cancer progression and diversity is likely to be advanced greatly by the multiomics investigation of single cells, as is our understanding of normal developmental and other disease processes.

Ongoing technological advances will see improvements in the coverage, sensitivity of multiomics approaches, as well the number of analytes that can be surveyed in parallel.

## Multiple Molecular Types in Cells

The cell is a natural unit of biology, whose type and state can vary according to external influences or to internal processes. In multicellular organisms, all cells are derived from a single zygote which, through regulated programmes of proliferation and differentiation, generates all of the diverse cell types that populate the organism. Dysregulation of these programmes in single ‘renegade’ cells can lead to diseases such as cancers [1], neurological disorders [2] and developmental disorders [3].

Sequencing technologies now permit genome [4], epigenome [5], transcriptome [6], or protein [7] profiling of single cells sampled from heterogeneous cell types and different cellular states, thereby enabling normal development and disease processes to be studied and dissected at cellular resolution. However, the sampling of just one molecular type from individual cells provides only incomplete information because a cell's state is determined by the complex interplay of molecules within its genome, epigenome, transcriptome and proteome. To more comprehensively understand and model cellular processes, new technologies are required to simultaneously assay different types of molecules, such as DNA and RNA or RNA and protein, to survey as much of the cellular state as possible.

Such multiomics approaches will enable, amongst other things, the generation of mechanistic models relating (epi)genomic variation and transcript/protein expression dynamics, which in turn should allow a more detailed exploration of cellular behaviour in health and disease. In this review, we discuss the developments, opportunities and challenges of sequencing technologies, which have enabled single-cell multiomics, and provide an outlook on future research and technological directions.

## Parallel Interrogation of Genomes and Transcriptomes

The ability to survey both the genome and the transcriptome of the same single cell in parallel will offer a number of unique experimental opportunities. Primarily, it would directly link the wild-type or modified genotype of a cell to its transcriptomic phenotype, which reflects, in turn, its functional state. Genomic variation in a population of cells could be associated with transcriptional variation, and molecular mechanisms that are causal of cellular phenotypic variation could be deduced without the potentially confounding effects of cell type heterogeneity. Second, single-cell genome sequences could be used to reconstruct a cell lineage tree that captures the genealogical record of acquired DNA mutations in the cells’ genomes over time; in parallel, the RNA sequences of these same cells would reflect the types and states of the cells. These phenotypically annotated lineage trees should enhance our understanding of the cellular properties and population architectures of heterogeneous tissues in health and disease.

Direct measurement of multiple molecular types in the same cell offers substantial advantage over the separate measurement of each molecular type in different cells. This is because relating molecules, for example, RNA in one cell versus DNA in another (or in a population of cells), is confounded by the cells’ potential differences in genotype (e.g., somatic variation in cancer), phenotype (e.g., cell cycle) or environment (e.g., cell–cell interactions). Consequently, although a single cell's genomic copy number can be inferred indirectly from single-cell RNA-sequencing (scRNA-seq) data 8, 9, only by applying multiomics approaches to one cell can its genotype–phenotype relationships be determined unambiguously.

**Figure 1:**
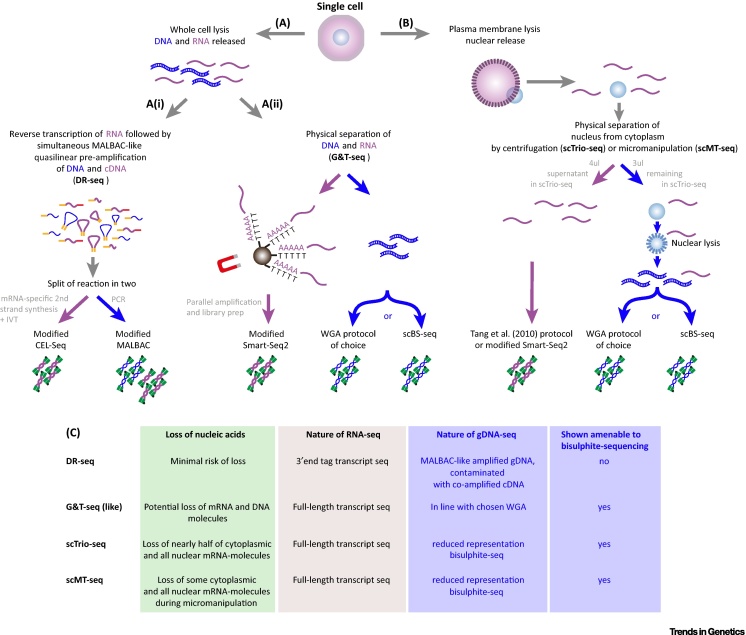
Experimental Approaches for DNA- and RNA-Sequencing of the Same Single Cell. (A) Following complete cell lysis, one has the option to follow principles of genomic DNA (gDNA)–mRNA sequencing (DR-seq) or genome and transcriptome sequencing (G&T-seq). (i) In DR-seq, lysis of the cell is directly followed by reverse transcription of RNA to synthesise single-stranded cDNA incorporating the 5′ T7 promoter. The gDNA and single-stranded cDNA are then amplified together in the same reaction by a quasi-linear approach, using principles of the multiple annealing and looping-based amplification cycles (MALBAC) protocol for single-cell WGA, after which the reaction is split. gDNA in one half of the reaction is further amplified by PCR, leading to co-amplification of contaminating cDNA, while in the other reaction cDNA-specific second-strand synthesis is followed by *in vitro* transcription (IVT) for further amplification of the mRNA. (ii) In G&T-seq, lysis of the cell is followed by physical separation of polyadenylated mRNA from DNA using oligo-dT-coated magnetic beads either manually or on a robotic liquid handling platform. Following separation, the mRNA is converted on the bead to cDNA and further amplified using a modified Smart-seq2 approach. The DNA of the same cell is precipitated and prepared for genome sequencing following a WGA method of choice or for methylome sequencing following bisulphite conversion and amplification. Alternatively, (B) following lysis of the cell membrane but not the nuclear membrane, the nucleus can be physically isolated from the cytoplasmic lysate of the cell. The latter contains the cytoplasmic mRNA molecules and can be used for the preparation of a RNA-seq library. In parallel, the nucleus containing the genomic DNA can be lysed and used for the preparation of genome sequencing or (reduced representation) DNA methylation sequencing libraries, as in single-cell methylome and transcriptome sequencing (scMT-seq) and single-cell genome, DNA methylome and transcriptome sequencing (scTrio-seq). (C) Comparison of pros and cons of current single-cell multiomics methods. scBS, single-cell bisulphite sequencing; WGA, whole-genome amplification.

Two complementary strategies have been developed that permit both genome and transcriptome sequencing from single cells (Figure 1, see Box 1 for information about single-cell isolation). In the first approach, gDNA–mRNA sequencing (DR-seq) [10] (Figure 1A), genomic DNA (gDNA) and mRNA present in a single cell's lysate are preamplified simultaneously before splitting the reaction in two for parallel gDNA [using a modified multiple annealing and looping-based amplification cycles (MALBAC) [11] approach] and mRNA library preparation (using a modified CEL-seq [12] approach) and subsequent sequencing. In the other approach, exemplified by genome and transcriptome sequencing (G&T-seq) 13, 14 (Figure 1A), mRNA is physically separated from gDNA using oligo-dT-coated beads to capture and isolate the polyadenylated mRNA molecules from a fully lysed single cell. The mRNA is then amplified using a modified Smart-seq2 protocol 15, 16, while the gDNA can be amplified and sequenced by a variety of methods 13, 14. The transcriptogenomics method [17] is based upon a similar principle of separation and parallel amplification. Separation of genome and transcriptome can also be accomplished using more gentle cell lysis procedures that dismantle the cellular but not the nuclear membrane (Figure 1B), allowing the intact nucleus to be separated from the cytoplasmic lysate; the nucleus can be used as a substrate for genomic [18] and epigenomic analysis 19, 20, while the cytoplasmic lysate can be used to perform mRNA profiling of the single cell. In addition to these methods, which apply microliter volume reactions, a microfluidic platform method using nanolitre reaction chambers that physically separates cytoplasmic mRNA from nuclear gDNA of the same single cell was described [14], which can be used for targeted amplicon sequencing of both molecular types.

Box 1 **Isolation of Single Cells**

Ensuring that a sample contains only a single cell remains technically challenging. The first key step is to generate a single-cell suspension. This varies considerably between tissue types and optimisation is required to ensure analysis of a viable, unbiased, cell population. When tissue complexity or handling prohibits intact cell isolation, suspensions of single nuclei can be prepared 68, 69. Single nucleus (epi)genomic and transcriptomic analyses have been demonstrated 19, 68, 69, and thus in principle solely nuclei may be used as input for multiomics approaches.

There are various approaches for isolating single cells from a suspension. Manual isolation – either using specialised pipettes or micromanipulation equipment – notably allows a single cell to be directly visualised during isolation. When all of a small number of cells are to be analysed – for example, daughter cells from a single cell division – this is often the most suitable option [70]. Nevertheless, it is by necessity low throughput.

FACS allows phenotypically distinct cells, and even nuclei, to be sorted into user-defined vessels and lysis buffers, thus enabling diverse single-cell and single-nuclei protocols to be applied at significantly higher throughput [68]. Index sorting [71] additionally allows direct linking of a single cell's phenotype (e.g., surface marker expression, DNA content) with multiomics analysis. However, large numbers of cells are required as input, and because the platform currently offers no opportunity to visualise sorted cells, care must be taken to identify and exclude cell doublets.

Microfluidics technologies that isolate single cells in individual capture sites and initiate nucleic acid amplification in nanolitre volumes have been widely applied in single-cell omics studies (e.g., Fluidigm C1 [72]). Once captured, cells can be visualised on the chip, confirming the presence of a single cell.

Advances in microfluidics approaches in which single cells are encapsulated within individual droplets prior to barcoded sequence library preparation (e.g., Drop-seq [73], inDrop 74, 75) allow tens of thousands of single cells to be investigated in parallel. However, these approaches rely on limiting dilution Poisson statistics for cell isolation, which result in a doublet rate dependent on the concentration of cells in the input material. Visual validation is not currently a component of these protocols.

Single cells can also be isolated using laser capture microdissection [76], which offers a unique opportunity to study cells in their topological context, although this has not yet been applied widely to multiomics analysis.

To achieve success, single-cell protocols need to maximise accuracy, uniformity and coverage when sampling a cell's available molecules. Minimising the loss, while maintaining the diversity and fidelity of information from a single cell, is a critical challenge in the development of multiomics approaches. The major advantage of avoiding *a priori* separation, as in DR-seq, is that it minimises the risk of losing minute quantities of the cell's genomic/transcriptomic material during any transfer steps, whereas the advantage of physical separation is that the cell's gDNA and mRNA are amenable to independent protocols of choice for further amplification and sequencing (Figure 1C). However, protocols that rely on physical separation of nucleus and cytoplasm 19, 20 are often dependent on manual isolation of the nucleus from each single cell and thus such methods, unless transferred to a microfluidics platform [18], may only be applicable in low-throughput settings.

## Linking Genomic and Transcriptomic Variation in Single Cells

**Figure 2:**
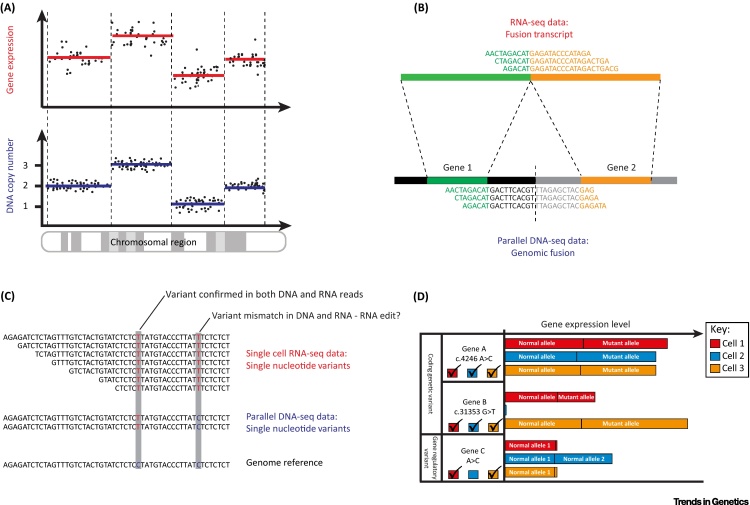
Integrative Genome and Transcriptome Sequence Analyses of Single Cells. Single-cell genotype–phenotype correlations are enabled by sequencing its DNA and RNA, including (A) investigating gene expression dosage effects resulting from DNA copy number alterations; (B) detecting the expression of fusion transcripts from DNA structural variation, permitting base-level reconstruction of both fusion transcript and the causative genomic lesion; (C) studying the expression of coding genomic variants – including allele-specific expression or the expression of an acquired single nucleotide variant – or observing RNA editing; and (D) examining the expression level of transcripts from genes mutated in their coding or noncoding genomic parts (e.g., a gene regulatory region), and thus determining the functional consequences of acquired genetic variation on the cell. We note that limitations of transcriptional profiling for inferring genomic variation include that (i) only genomic variants within or encompassing expressed genes in the cell can be represented in the single cell's RNA-seq data – that is, nontranscribed genomic variation may not be inferred from the cell's RNA-seq data alone; (ii) artefacts resulting from whole-transcriptome amplification (and whole-genome amplification) should be taken into account when inferring genomic variation; and (iii) transcriptional profiles can be less predictive of genomic variation when read coverage is limited.

The first-generation methods for multiomics single-cell sequencing – DR-seq and G&T-seq in particular – demonstrated how genomic variation among a population of single cells can explain transcriptomic variation. Both methods were applied to reveal, for the first time, the direct association between (sub)chromosomal copy number and gene expression in the same single cell (Figure 2A). DR-seq demonstrated a positive correlation between large-scale DNA copy number variation in the genome and gene expression levels in individual cells. Furthermore, these data indicated that genes with low DNA copy number tend to generate transcripts with noisier expression levels [10]. G&T-seq was applied to human breast cancer and matched normal lymphoblastoid cell lines, as well as to primary cells from eight-cell stage mouse embryos and human inducible pluripotent stem cell-derived neurons derived from individuals with either a disomy or trisomy for chromosome 21. Data from these G&T-seq experiments further confirmed the relationship between (sub)chromosomal copy number and expression level of genes located within DNA copy number variable regions in single cells [13].

These approaches also allow the functional consequences of *de novo* structural variants to be investigated in single cells. In cancer, structural DNA rearrangements can translocate gene regulatory elements to the vicinity of other genes thereby perturbing their expression, or may result in novel fusion genes, which contribute to the overall progression of the disease. With G&T-seq, the full length of the mRNA molecule is preserved during amplification (Figure 1C), which enables the detection of expressed fusion transcripts either by assembling Illumina short reads or as long reads using the Pacific Biosciences RSII sequencer [13]. The concurrent availability of a matched genome sequence from the same single cell allows the causal genomic fusion to be validated and mapped to single base resolution, in parallel with the ability to detect genome-wide dysregulation of gene expression associated with a structural rearrangement (Figure 2B).

DR-seq [10], G&T-seq [13] and the method described by Li *et al.* [17] all have potential to detect single nucleotide variants (SNVs) in matched single-cell genomes and transcriptomes. This enables, if the transcript carrying the variant allele is expressed, confirmation of the detection of SNVs in two readouts from the same cell. Where genome coverage is sufficient to detect both alleles of an expressed gene, it would also be possible to extend this analysis to consider allele-specific expression, with the cell's own genome as a reference. Furthermore, the comparative analysis of genome and transcriptome sequencing data from the same single cell should enable the detection of RNA editing events, again using the cell's own genome as a reference (Figure 2C). The availability of both DNA and RNA sequencing data from the same cell also has clear potential to enable the detection of expressed, coding mutations in populations of single cells (Figure 2D, upper and middle panels) as well as the study of acquired expression quantitative trait loci, whereby *de novo* genetic variants in, for instance, gene regulatory elements of single cells may affect the expression of the gene(s) under the control of this element, altering the cell's transcriptomic cell state (Figure 2D, lower panel), or how newly acquired genomic variants may alter the splicing or reading frame of a transcript in a cell.

However, limitations in whole-genome amplification mean that detection of all classes of variants currently cannot be achieved comprehensively and with complete accuracy in every single cell 4, 21. All whole-genome amplification approaches result in frequent allelic and locus dropouts – in which, respectively, either one or both alleles of a sequence are not detected leading to false-negative calls and it is likely that physical separation or manipulation of gDNA in multiomic assays can exacerbate the levels of dropout observed. Furthermore, all polymerases have a baseline error rate, and thus base misincorporation errors occur during amplification of both DNA and RNA leading to false-positive SNV calls.

Additional limitations exist in whole-transcriptome amplification approaches. Reverse transcriptase and subsequent polymerase-based amplification steps also have potential to introduce biases in representation in the data. In single-cell whole-transcriptome amplification, it is estimated that only 10–40% of the original mRNA molecules from a cell are represented in the final sequencing library 22, 23, and again, it is feasible that either parallel amplification or physical separation of DNA and RNA could potentially reduce this level even further (Figure 1C).

Improvements in single-cell amplification and library preparation, in addition to the optimisation and development of technologies for separation of different analytes from the same cell, are an ongoing area of research in multiomics protocol development, and key technical challenges must be met to enable the full potential of the approach (see Outstanding Questions).

## Multiomics Analysis of Single Cells in Cancer

Multiple types of mutation can be introduced over the trillions of cell divisions that occur during the lifespan of a multicellular organism – from SNVs and interchromosomal or intrachromosomal rearrangements to gains or losses of whole chromosomes or even entire genomes 2, 21. Current multiomics approaches stand ready to disclose the functional consequences of those acquired mutations and how they contribute to the spectrum of normal phenotypic variation, developmental and neurological disorders as well as other diseases. The single-cell genotype–phenotype correlations that these methods provide enable unique insights into diverse biological and disease processes, particularly for cancer, in which somatically acquired genomic diversity and its transcriptional consequences are key components of the origin and evolution of the disease.

**Figure 3:**
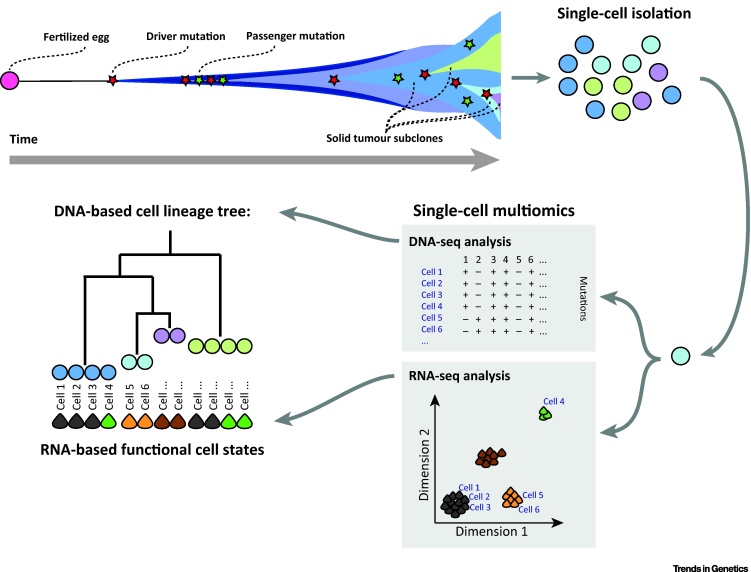
Cell Lineage Trees Depict the Genealogical Record of Acquired DNA Mutations Overlaid with the Transcriptomic Phenotypes of the Same Cells. Cancers arise due to the acquisition of driver mutations (red stars) in a single cell, resulting in a clonal expansion of that cell (blue droplet). During this expansion, more driver mutations can accumulate giving rise to tumour subclones (green, light blue and purple droplets) with common and unique DNA mutations. Single-cell multiomics on a heterogeneous population exposes both the acquired DNA mutations per cell by DNA-seq analyses and the transcriptomic cell type and state of each cell by dimensionality reduction techniques on RNA-sequences of the same cells. The single-cell DNA mutation matrix can reconstruct the cell lineage tree, which can then be overlaid with transcriptomic states of the same cells, disclosing the gene expression of profiles of, for example, tumour subclones.

Single-cell multiomics approaches can uniquely relate acquired genomic variation with changes in cellular function and transcriptional phenotype in cancer (Figure 3). Furthermore, these approaches may contribute to the understanding of cellular mechanisms of resistance to cancer therapies – it is conceivable that genetically similar cells belonging to a particular subclone may develop distinct transcriptional cell states resulting in functional dissimilarities and differential drug responses. By determining the genomic and transcriptional states of such cells in parallel, it may be possible to reveal the transcriptional signature – and potentially molecular targets – which regulate the diversity in responsiveness to therapy.

One of the principal applications of single-cell genome sequencing is the establishment of lineage trees – or phylogenies – of cancer evolution. Theoretically, the cell lineage of a cancer can be reconstructed by considering the degree by which cells share somatic variants, each inherited from a common ancestral cell in which it first arose [24]. Following reconstruction of the subclonal genomic lineage of a cancer to single-cell resolution, single-cell multiomics approaches can be used to annotate the lineages within the tumour with transcriptomic cell states (Figure 3).

DNA-based cell lineage trees annotated with transcriptomic cell state information will not only be of use in understanding the extent, nature and biology of genomic–transcriptomic cellular heterogeneity in cancer over the course of treatment, but also in revealing the cellular architecture and developmental history of organs in healthy organisms. Single-cell genomics has revealed a spectacular degree of genetic variation in the human brain – ranging from low-frequency aneuploidies to high-frequency copy number variants and SNVs, even in young individuals 25, 26. It is likely that any multicellular organism comprises a mosaic of genomes, with mutations acquired throughout its development disclosing the cellular lineage [24]. By extending these phylogenetic studies to incorporate a multiomics approach, it becomes possible not just to infer the cellular phylogeny of an organism or diseased tissue, but to annotate that phylogeny with an atlas of transcriptional phenotypes for the individual cells. Integrating lineaging approaches within current efforts to generate cell atlases for whole organisms will allow the phylogenetic relationships of the cells to be inferred, which in turn may contribute to the understanding of tissue and organismal development.

## Linking Epigenetic and Transcriptomic Variation in Single Cells

DNA methylation at the carbon-5 position of cytosine bases, primarily in a CpG dinucleotide context, is a common correlate of gene expression variation in mammals [27]. Nevertheless, to explore the causal or consequential basis for this correlation, it is necessary to measure both DNA methylation and transcript abundance for the same single cells sampled from dynamic and heterogeneous cell populations. Approaches that integrate single-cell DNA methylation analysis, including single-cell bisulphite sequencing (scBS-seq) [28] and reduced representation bisulphite sequencing (scRRBS-seq) [29], with single-cell RNA-seq have recently been developed. In contrast to scBS-seq, scRRBS-seq first digests the cell's gDNA with a methylation-insensitive restriction enzyme (e.g., *Msp*I) prior to bisulphite treatment, which allows enrichment for CpG-rich DNA sequences, giving a reduced but representative overview of DNA methylation in the cell's genome. All currently described DNA methylation and RNA transcript abundance methods require a physical separation and then amplification of gDNA and mRNA as described earlier (Figure 1), and exploit the conversions of unmethylated cytosines to uracils by bisulphite treatment: these converted bases are detected as thymine bases following amplification and sequencing, while methylated cytosines are not altered.

The first such method, single-cell methylome and transcriptome sequencing (scM&T-seq) [30], is an extension to the G&T-seq protocol in which mRNA is captured, amplified and sequenced as before. However, the isolated gDNA of the single cell undergoes bisulphite sequencing [28], rather than whole-genome amplification, allowing parallel analysis of genome-wide DNA methylation and transcriptome-wide gene expression from the same single cell.

Subsequently reported methods involved the physical separation of the cytoplasmic RNA and the nuclear DNA before parallel amplification of cDNA and bisulphite-treated DNA (Figure 1B). In scMT-seq [20], the cell is gently lysed, and the nucleus collected by microcapillary picking. The mRNA in the lysate is then amplified by a modified Smart-seq2 protocol 15, 16, while the genome is subjected to a modified scRRBS protocol. In single-cell genome, DNA methylome and transcriptome sequencing (scTrio-seq) [19], the nucleus and cytoplasm are separated by centrifugation, the transcriptome is then amplified as described in 31, 32 and the scRRBS approach is applied to the gDNA.

**Figure 4:**
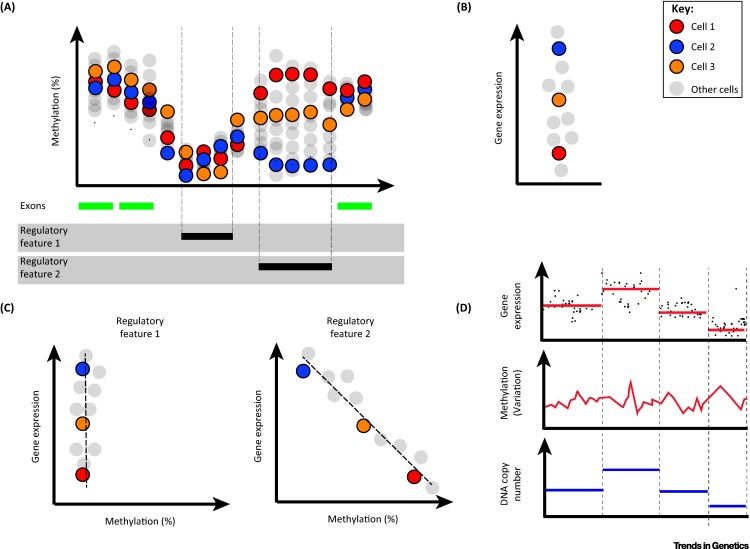
Integrative Epigenome and Transcriptome Sequence Analyses of Single Cells. (A) Gene regulatory elements, in particular, can show homogeneous (Regulatory Feature 1) or heterogeneous (Regulatory Feature 2) methylation states in populations of cells. (B and C) The variance in DNA methylation across cells at a particular genomic locus may be coupled or uncoupled to variance in expression of nearby gene(s) across the cells. Parallel single-cell analysis of DNA methylation and gene expression allows the correlation of expression measurements with methylation at various potential regulatory sites. (D) From single-cell DNA-bisulphite sequences, it is also possible to compute DNA copy number profiles (e.g., scTrio-seq), allowing investigation of correlations between DNA numerical alterations, DNA methylation states and transcriptomic phenotypes across single cells in a population. scTrio-seq, single-cell genome, DNA methylome and transcriptome sequencing.

All three methods allow for the consideration of whether the degree of DNA methylation of different functional elements in the genome – e.g., gene bodies, promoters, enhancers – reflects the expression levels of genes in single cells (Figure 4A–C). The scRRBS approaches detected approximately 480 000–1 500 000 CpG sites, while scM&T-seq, which utilises genome-wide bisulphite conversion, had a greater coverage of approximately 4 500 000 sites (approximately 25% coverage) from a similar depth of sequencing per cell 19, 20, 30.

Uniquely, the scTrio-seq approach [19] computationally mines both the DNA methylation and copy number states from scRRBS sequencing libraries, and in parallel measures cytoplasmic transcript levels from the same single cell (Figure 4D). In principle, DNA copy number landscapes could also be mined from the methylome sequences of scM&T-seq and scMT-seq data but this has yet to be demonstrated.

In addition to bisulphite treatment, one other approach has been developed for targeted DNA methylation profiling of multiple loci in single cells. This involves the restriction digestion of single-cell DNA with methylation-sensitive enzymes followed by multiplexed quantitative real-time PCR with primers flanking the restriction sites in a microfluidics device 33, 34. The technology was recently combined with RT-quantitative PCR (qPCR)-based targeted gene expression and sequencing-based targeted genotyping of genomic loci [35], enabling single-cell analysis of genotype, expression and methylation (scGEM). In contrast to bisulphite-based DNA methylation assays that are prone to stochastic dropouts, scGEM enables a more reproducible assessment of methylation status at specific sites across cells.

Beyond methylation, the repertoire of approaches available to study single-cell epigenomics continues to expand [36]. The recent demonstration that genome-wide hydroxymethylation can be measured in a single cell [37], along with the emergence of approaches that survey open chromatin (assay for transposase-accessible chromatin using sequencing [38] and single-cell DNase sequencing [39]), chromatin conformation (Hi-C [40]), or DNA- or chromatin-binding proteins (ChIP-seq [41], DNA adenine methyltransferase identification (DAM-ID) [42]), opens up new opportunities to couple a diverse array of single-cell epigenomic methods with parallel transcriptomic analysis. Epigenomic mechanisms are central to the regulation of gene expression and the emergence of functional diversity across cells with identical genomes; and as a consequence, parallel study of the epigenomes and transcriptomes of single cells is fundamental to understanding cellular identity, cellular function and phenotypes that are not predictable by genotype alone.

## Relating Expression of RNA and Protein in Single Cells

Single-cell proteomics methodologies, while still limited in survey breadth when compared to genomics approaches, are developing rapidly. Currently, the most widely applied single-cell proteomics approaches rely on targeting specific proteins using tagged antibodies. Fluorescence-based detection of protein by fluorescence-activated cell sorting (FACS) or fluorescence microscopy, as well as single-cell Western blotting [43], allow protein detection in single cells with a low level of multiplexing (approximately 10–15 proteins in total), whilst more highly multiplexed targeted approaches, including the use of oligonucleotide-labelled antibodies followed by qPCR 44, 45, metal-tagged antibodies followed by mass cytometry 46, 47 and single-cell mass spectrometry [48] are emerging. However, these initial methods are still limited to the detection of tens to hundreds of proteins per cell.

Nevertheless, the incorporation of proteomic and transcriptomic analysis into a single multiomics approach offers the exciting possibility to explore the dynamics of RNA and protein abundance in the same single cell. The most straightforward approaches employ indexed FACS of single cells to relate immunofluorescent signals of a single protein, or a small number of proteins, with their corresponding transcripts’ levels in the same cells [49]. More advanced methods have used FACS- and imaging-based approaches to simultaneously measure mRNA and protein 50, 51.

One approach to simultaneously read out protein and transcript abundance from the same single cell is to employ a proximity extension assay (PEA) [45] in parallel with RNA detection. In a PEA, two antibodies that recognise different epitopes of the same protein are tagged with complementary oligonucleotides. These oligonucleotides hybridise when in sufficient proximity (i.e., bound to the same molecule), allowing mutual priming and extension and hence generating a unique sequence that can be further amplified and detected by qPCR. Thus, the protein signal detected in a single cell is transformed into a nucleotide signal that can be interpreted by qPCR and, as has been demonstrated in bulk samples, high-throughput sequencing [52]. By incorporating unique sequences with every PEA antibody pair, a substantial number of proteins (>70) can be investigated in parallel with the level of the corresponding transcripts.

**Figure 5:**
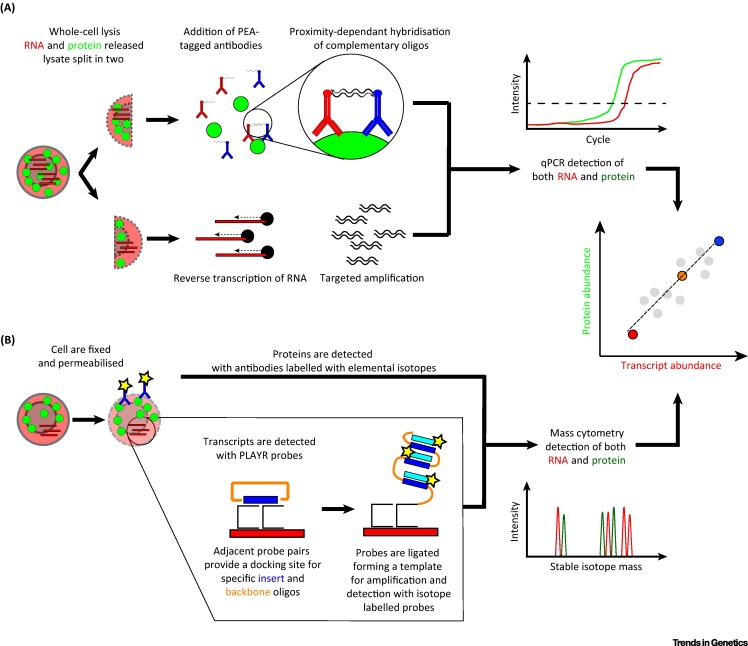
Experimental Approaches for Integrative Transcript and Protein Analyses of Single Cells. (A) The lysate of a whole cell can be split into two separate reactions: one for measuring abundance of specific proteins by quantitative PCR (qPCR)-based proximity extension assay (PEA) and one for quantifying transcripts of specific genes by qPCR on cDNA. (B) Cells are fixed and permeabilised, and specific transcripts and proteins are marked for mass cytometry quantification. To this end, transcripts are first hybridised with proximity ligation assay for RNA (PLAYR) probes, and following ligation of the insert (blue) and backbone (orange) probes, this circle is amplified by rolling circle amplification and the multiple insert sequences per transcript are hybridised with mass cytometry-compatible probes. Specific proteins are quantified using antibodies conjugated to distinct heavy metals enabling mass cytometry measurement. Both methods then enable the correlation of protein and transcript abundance from the same single cells. Here, a positive correlation is shown, although it is expected that this correlation would vary depending on the dynamics of transcript and protein expression.

One such method (Figure 5A) involves splitting single-cell lysates and performing qPCR to detect the transcript of interest in one half, while the other half is incubated with a PEA [53]. Using this method, the correlation of abundance of transcripts and proteins from 22 genes in single cells could be investigated. This approach is, in principle, compatible with existing single-cell RNA-seq methods, and thus could be employed to generate data from a small number of proteins of interest together with transcriptome-wide sequencing data. More recently, parallel PEA-based detection of protein and targeted RNA analysis has been demonstrated in a single series of reactions on the Fluidigm C1 platform [54]. Rather than splitting cell lysates, PEA extension and reverse transcription are performed in parallel and the products are measured by qPCR, which in this study enabled parallel measurement of 38 proteins and 96 transcripts.

Proximity ligation assays utilise a similar method [44], but rely on the ligation, rather than hybridisation, of two antibody-conjugated oligonucleotides brought into proximity on the same protein target. This approach has successfully been integrated into a protocol for targeted parallel analysis of DNA, RNA and protein from the same single cell [55] and translated to a droplet digital PCR platform to enable parallel measurement of a single protein and its corresponding transcript [56]. Recently, a proximity ligation assay for RNA (PLAYR; Figure 5B) was developed that allows the quantification of multiple specific transcripts by mass cytometry in thousands of single cells per second [57]. As the method allows metal-tagged antibody staining in parallel, simultaneous proteomic and transcriptomic measurements for ten(s) of genes can be made for the same single cell using mass cytometry.

Multiomics techniques, which utilise antibodies for protein detection will always, however, be limited by the availability of high-affinity reagents. Consequently, studies investigating the relative dynamics of transcript and protein expression will largely remain biased towards the selection of systems for which suitable antibody panels are available; those dependent on mass cytometry will also be limited by the quality and availability of suitable metal ions for detection. Enabling single-cell genomic and transcriptomic approaches, which are also compatible with increasingly sensitive mass spectrometry-based approaches 48, 58, could remove this restriction, which would allow a broader investigation of proteomic dynamics as part of a multiomic single-cell readout. However, without significant increases in sensitivity, it seems that whole proteome characterisation of single cells by any approach remains elusive.

## Towards ‘Omniscience’ – How Much Can We Know about a Single Cell?

**Figure 6:**
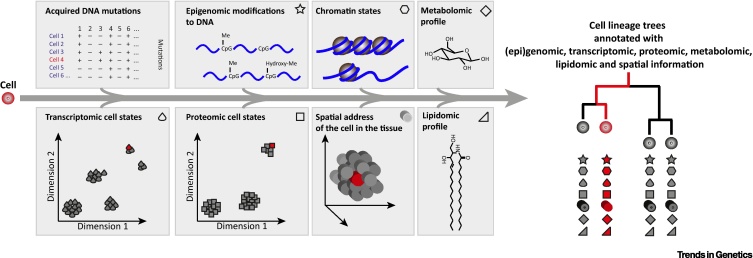
Key Figure: Spatial Omni-Seq of Single Cells

While there is much we can learn from surveying each of the genome, epigenome, transcriptome or proteome of single cells in turn, predictive modelling of cellular dynamics will require the integration of these – and more – data sets from the same single cell (Figure 6, Key Figure).

Nevertheless, the limitations in sensitivity of single-cell genomics approaches remain a key challenge. For multiomics approaches including genomic and epigenomic analyses, allelic and locus dropout is common and, as such, base-level events are often impossible to detect consistently and comprehensively. The transcriptome obtained from most single-cell RNA-seq methods is generally restricted to a portion of the polyA+ mRNA present in the cell and will not, for example, detect mature microRNA molecules and consequently an entire regulatory layer within the cell will not be observed. Methods for total RNA [59] and small RNA [60] sequencing from single cells are emerging, and integrating these approaches into existing multiomics frameworks will enable a more comprehensive overview of the transcriptional phenotype of a single cell.

Single-cell proteomics approaches remain limited to multiplexed measurements of a low number of proteins per cell, thus their integration into multiomics approaches in the near future will be targeted very specifically at systems for which panels of reliable antibodies are available. In addition, other molecular classes such as lipids and metabolites may reflect important aspects of cellular state, and although methods for single-cell metabolomics are emerging [61], integrating these approaches with other omics technologies will bring unique challenges.

It does, however, appear that progress will be made to increase the number of parallel observations that can be made from a single cell towards ‘omni-seq’: a complete molecular readout of the state of the cell. Despite the enormous technical challenges and technological development required to address the current limitations of single-cell sequencing approaches, this goal represents a worthwhile aspiration because of the lessons to be learnt in its pursuit, as well as the complex and unique observations that can be made from data that remain far from complete. Indeed, a key challenge for the technology will be the computational processing and integration of these data.

## Concluding Remarks

In some form, however, the integration of genomic, epigenomic, transcriptomic and proteomic data appears to us a realistic prospect. In part, this is because such an approach will benefit from rapidly developing sequencing technology, which can interpret more than one analyte in parallel – for example, Pacific Biosciences and nanopore sequencers (e.g., Oxford Nanopore) can detect DNA modifications (e.g., methylation 62, 63) in addition to sequencing native DNA molecules. Both technologies are also capable of direct RNA sequencing 64, 65, and nanopores have additionally been demonstrated to detect protein modifications [66] and other analytes (e.g., peptides, microRNAs and small molecules [67]). Thus, we anticipate that future developments in molecular sequencing and detection approaches may provide the critical advances that expand and refine single-cell multiomics approaches to the point where comprehensive atlases of cell state and lineage can be generated for cellular systems, ranging from tissue microenvironments to whole organisms (see Outstanding Questions).

Outstanding Questions How can single-cell information be exploited by computational models to more accurately predict cellular phenotypes and their dynamics?

To what degree are molecules altered by methods that isolate single cells from their physiological context?

Can single-cell multiomics methods be combined with spatial measurements, perhaps even in real time?

Can highly accurate single-cell genome sequences be attained given the diversity of artefacts – that are often indistinguishable from genuine genetic variants – introduced by current amplification methods?

Can the conversion rate of single-cell mRNA molecules to amplifiable and sequenceable cDNA molecules be improved and extended to simultaneous measurement of small noncoding RNAs?

What is the optimal computational approach to calling DNA variants jointly, using sequence data from multiple molecular modalities?

What is the upper limit to the fraction of all molecules in a cell whose presence can be detected experimentally?

In particular, given that single-cell proteomics is currently of relatively low throughput, will it ever be feasible for abundance measurements to be made for many hundreds to thousands of proteins from the same single cell?
